# Supplementary material for: A rare variant analysis framework using public genotype summary counts to prioritize disease-predisposition genes
Source: Nat Commun. 2022 May 11;13:2592. doi: 10.1038/s41467-022-30248-0 (PMC9095601; doi:10.1038/s41467-022-30248-0)
Supplement: Supplementary file 1 — Supplementary Information [file 41467_2022_30248_MOESM1_ESM.pdf]

Supplementary Methods

Efficient calculation of coverage summary information for subsequent coverage-based filtering

We used samtools[1] to calculate the coverage depth at all qualified positions. We used the aligned bam input, with the minimal base quality ≥10 and the minimal mapping quality ≥20, following the coverage calculation in gnomAD[2]. Each sample was processed independently; therefore, this step can be easily parallelized for each sample. Then we aggregated the coverage data of each sample incrementally to update the summary information, e.g., the total coverage and the total number of samples with coverage depth $\geq x$, where x is from the set $\{1, 5, 10, 15, 20, 25, 30, 50, 100\}$. At the end, the summed numbers were divided by the total number of samples to calculate the average coverage and the proportions of samples greater than or equal to each coverage threshold. Instead of simultaneously calculating the depth at each position for all samples, e.g., using GATK *DepthOfCoverage*, this design of calculating the coverage summary information can be easily scaled-up to a large number of samples with constant memory requirement and easy parallelization. This part is implemented in Python and bash.

Variant blacklist from the gnomAD

The publicly available gnomAD database has a very large sample size and has undergone comprehensive quality control (QC) analysis[2]. For example, it employs a random forest-based and allelic-specific QC to achieve better performance than VQSR. Therefore, we used gnomAD filtering status (gnomAD v2.1) for further QC to remove likely false positives. The following 3 types of variants were excluded: 1) those that failed in both gnomAD whole-exome sequencing (WES) data and whole-genome sequencing (WGS) data; 2) those that failed in one platform and had no variant in the other; and 3) those with an alternate allele frequncy (AF) that differed substantially between WES and WGS. The last was done by testing the null hypothesis that the AF is the same within the WES and WGS in each of the five ethnicities in gnomAD (nfe, afr, amr, eas, fin). The test was done both individually within each ethnicity using a FET if not feasible, a chi-square test was used) and a combined test using a CMH test. Considering the potential differences in population structure between WES and WGS, we required that the p-value from the CMH test be <1e–15. Also, for at least one population, the p-value of the individual test should be <1e–10, and the ratio between AFs from the WES and WGS should be >1.2/1 or <1/1.2, or the absolute AF difference should be ≥0.15.

Principal component analysis and relatedness estimation

We used high-quality variants to calculate the principal components (PCs) and estimate the relatedness. Specificly, we used plink v1.9[3,4] and bcftools v1.9[5] to keep high-quality variants

with GQ ≥20 and DP ≥10. Then we filtered variants with missingness ≤0.01 and MAF ≥0.001. Samples with missingness >0.25 were also removed. Variants were pruned using plink with the option "--indep-pairwise 1000 50 0.1." Then we used *pcair* and *pcrelate* from the R package GENESIS v2.4.0 [4] to calculate the PCs and estimate the relatedness.

Simulation to assess performance of FDR control methods accounting for discrete counts

We simulated 10 or 50 causal genes under the alternative hypothesis and 20,000 genes under the null hypothesis. We set the number of cases to 300 and the number of controls to 50,000. We used the Beta(1, 10,000) distribution to simulate the frequency of pathogenic mutations in one copy of a gene and only kept the rare frequencies, specifically $<5\times10^{-4}$. The total number of samples with pathogenic mutations was then set by the simulated frequency multiplied by twice the total number of cases and controls. For the alternative hypothesis, we used Fisher's noncentral hypergeometric distribution to simulate the number of samples with the pathogenic mutations using the R package *BiasedUrn*[6]. For the null hypothesis, we used the hypergeometric distribution to simulate the number of samples with the pathogenic mutations. Different odds parameters of 40, 60, and 80 were used. In total, there were six different parameter combinations for the number of causal genes and odds parameters. We compared six different methods adjusting for multiple testing. These included two adopted resampling based FDR control methods: RBH_P and RBH_UL. respectively. We also included the BH method[7], and BH_T2 and BH_T3, which first filtered the genes with less than 2 or 3 samples with pathogenic mutations and then applied the BH method to the rest of the genes. We also included a method ADBH.sd recently developed for FDR control of FET[8]. The empirical FDR was the proportion of false positives among detected or 0 if none was detected. The empirical power was the proportion of true positives detected among all true positives. In total, 100 replications were performed, and the final average FDR and power were reported.

Type I error and power simulation for LD detection

We simulated six groups of summary counts, with the number of haplotypes being 2*e*4, 2*e*4, 5*e*3, 5*e*3, 3*e*4, 3*e*4, close to the sample size of stratified groups in the nfe population in gnomAD. The AFs of both variants were set to 0.001, 0.005, or 0.01. The odds ratio was set to 1 for evaluating the type I error and 10, 100, 1*e*3, 1*e*4, or 1*e*5 for evaluating the power. The HWE was assumed, and 1,000 replicates were simulated to estimate the type I error and power. One-sided test with alternative hypothesis that the odds ratio > 1 was performed. The nominal p-value threshold was set to 0.05. We also evaluated the type I error and power when genotypes were observed. We simulated 110,000 haplotypes summed into genotypes per two haplotypes and set the odds ratio to 1, 2, 5, 10, 20, or 50 for evaluation of type I error and power. Two-sided test was used because the function *ld* from the package snpStats only returns the two-sided test result.

Simulation of sample-count estimation including independent and correlated variants

To show that identifying high-LD rare variants can improve sample-count estimation based on summary counts, we simulated seven variants: the first two and the last two were fully correlated and the rest were independent, assuming the HWE. The number of individuals was set to 50,000. For each simulated data, there were five variants with AF = p, including two fully correlated variants, two fully correlated variants with AF = $2p$, and one variant with AF = $p/2$. The parameter p ranged from 0.01 to 0.001, each with three replicates in the simulated data sets. The root mean square error was calculated between the estimated counts and the ground truth. We assumed the frequencies of genotypes was observed in the estimation. We compared CoCoRV with TRAPD on count estimation. The method used in TRAPD overestimated the counts in controls to be conservative in the association test results when using a one-sided FET. For the dominant model, TRAPD added all qualified counts of each variant in a gene; for the double-heterozygous model (called compound heterozygous in Guo *et al.*[9]), the sum of the frequencies of heterozygous genotypes among all variants was squared and then multiplied by the total number of controls; for the recessive model, TRAPD added the counts of homozygous alternate genotypes and the counts from the double-heterozygous model.

Data processing and comparison between jointly called full genotype–based and summary count–based analysis

We used the two pediatric cancer cohorts (CNS and ALL) and our constructed in-house controls to compare the concordance between analyses using jointly called full-genotype data and that using separately called summary counts. All sequencing data were remapped using BWA v0.7.12[10] to the reference genome GRCh37-lite. We used GATK v3.7[11] to generate gVCF files for each sample. Three jointly called genotype data sets were generated: 1) full genotype data with all cases and controls jointly called, 2) in-house controls jointly called, and 3) case cohorts jointly called. The variants were normalized, and the multiallelic variants were decomposed into biallelic variants. We followed the GATK3 best practice to apply VQSR on each data set to QC variants. We only keep high quality genotypes which satisfied DP $\geq$10, GQ $\geq$20 and the allele fraction $\geq$0.2 and $\leq$0.8 for heterozygous genotypes using bcftools, the same as that used in gnomAD's QC process. We used the functions *pcair* and *pcrelate* from the R package GENESIS v2.4.0[12] to calculate the PCs and estimated the relatedness from the cases and controls by using the joint genotype calls. After excluding related samples and considering individual sample missingness, there were 8,175 samples in the constructed controls (5,602 from ADSP and 2,573 from the 1,000 Genomes Project). The CNS cohort had 336 cases and the ALL cohort had 958 cases.

Supplementary References

1 Li, H. *et al.* The Sequence Alignment/Map format and SAMtools. *Bioinformatics* **25**, 2078-2079, doi:10.1093/bioinformatics/btp352 (2009).

2 Karczewski, K. J. *et al.* The mutational constraint spectrum quantified from variation in 141,456 humans. *Nature* **581**, 434-443, doi:10.1038/s41586-020-2308-7 (2020).

3 PLINK 1.9, www.cog-genomics.org/plink/1.9/.

4 Chang, C. C. *et al.* Second-generation PLINK: rising to the challenge of larger and richer datasets. *Gigascience* **4**, 7, doi:10.1186/s13742-015-0047-8 (2015).

5 Danecek, P. *et al.* Twelve years of SAMtools and BCFtools. *Gigascience* **10**, doi:10.1093/gigascience/giab008 (2021).
6 Fog, A. BiasedUrn: Biased Urn Model Distributions. R package version 1.07. (2015).
7 Benjamini, Y. & Hochberg, Y. Controlling the False Discovery Rate: A Practical and Powerful Approach to Multiple Testing. *Journal of the Royal Statistical Society Series B* **57**, 289-300 (1995).
8 Döhler, S., Durand, G. & Roquain, E. New FDR bounds for discrete and heterogeneous tests. *Electronic Journal of Statistics* **12**, 1867-1900 (2018).
9 Guo, M. H., Plummer, L., Chan, Y. M., Hirschhorn, J. N. & Lippincott, M. F. Burden Testing of Rare Variants Identified through Exome Sequencing via Publicly Available Control Data. *Am J Hum Genet* **103**, 522-534, doi:10.1016/j.ajhg.2018.08.016 (2018).
10 Li, H. & Durbin, R. Fast and accurate short read alignment with Burrows-Wheeler transform. *Bioinformatics* **25**, 1754-1760, doi:10.1093/bioinformatics/btp324 (2009).
11 Poplin, R. *et al.* Scaling accurate genetic variant discovery to tens of thousands of samples. *bioRxiv* (2018).
12 Gogarten, S. M. *et al.* Genetic association testing using the GENESIS R/Bioconductor package. *Bioinformatics* **35**, 5346-5348, doi:10.1093/bioinformatics/btz567 (2019).

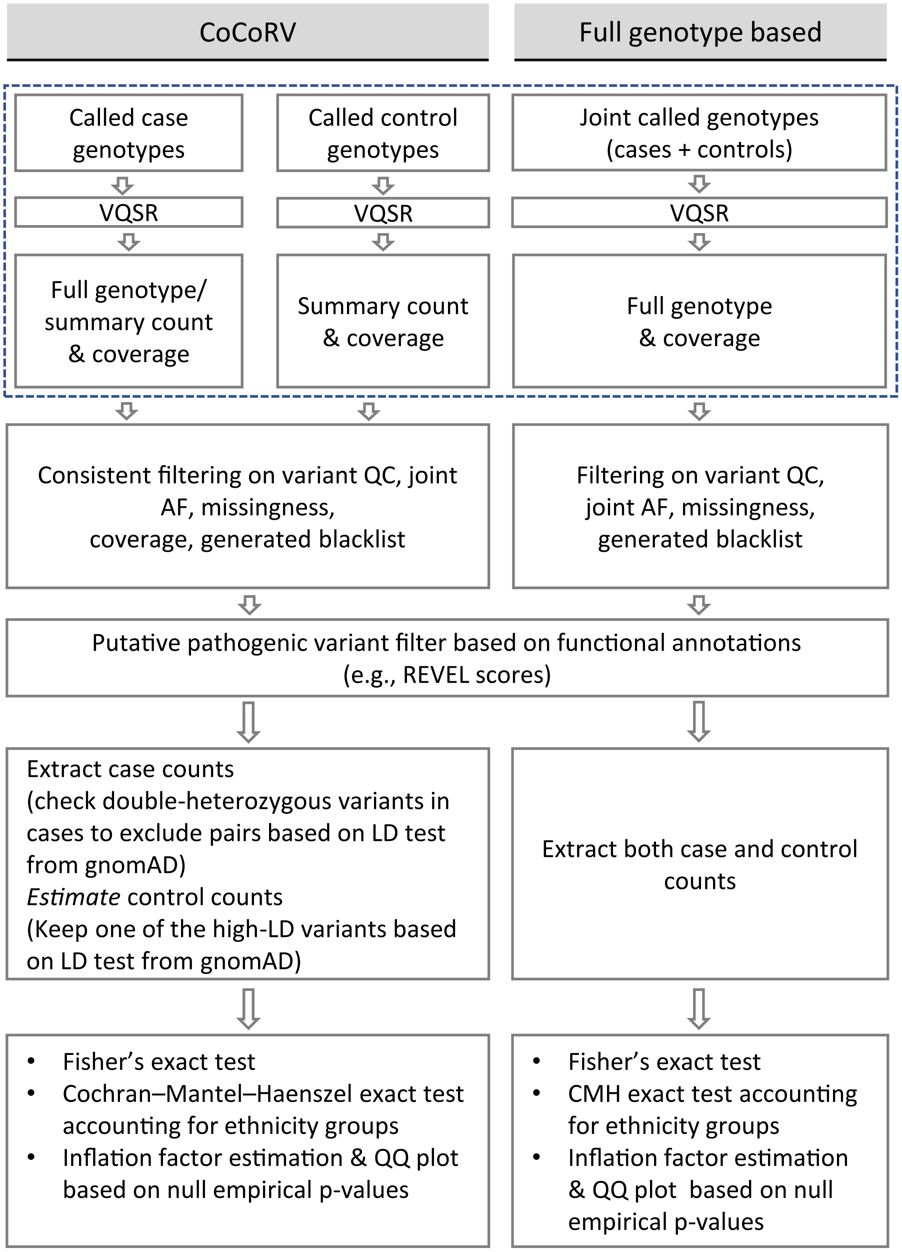

Supplementary Figure 1. Schematic diagrams comparing the proposed CoCoRV framework using summary counts and jointly called full genotype–based analyses. The left panel shows the CoCoRV framework using summary counts of separately called control cohort. The right panel shows the processing framework when cases and controls are jointly called. The diagrams within the dashed box show how data are generated differently for each analysis framework.

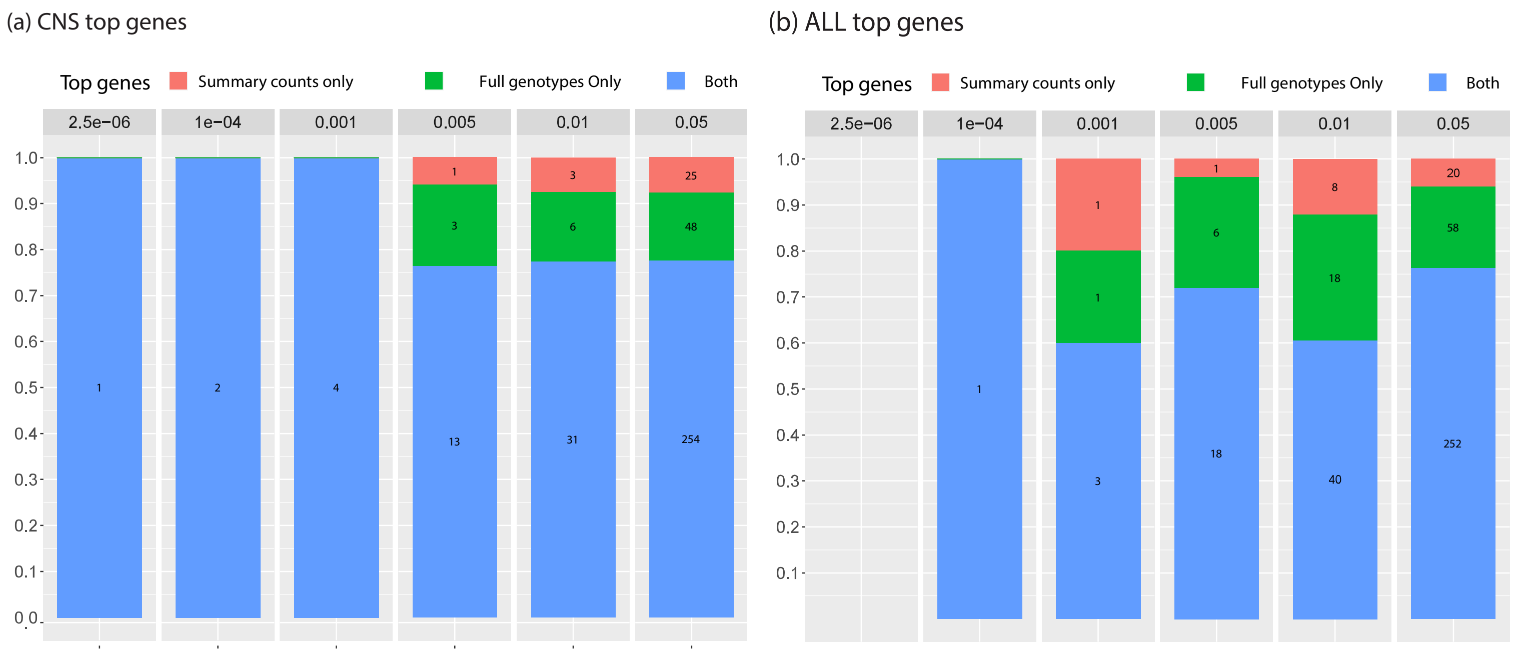

Supplementary Figure 2. Comparison of top genes between analyses using jointly called full genotypes and separately called summary counts using CoCoRV with the CMH exact test stratifying samples by ethnicities. The bar heights show the percentage and the numbers within each bar show the absolute number of genes. The p-value thresholds used are shown at the top. Results are based on analyses of the CNS (**a**) or ALL (**b**) cohort. All p-values are raw p-values from the two-sided CMH exact test.

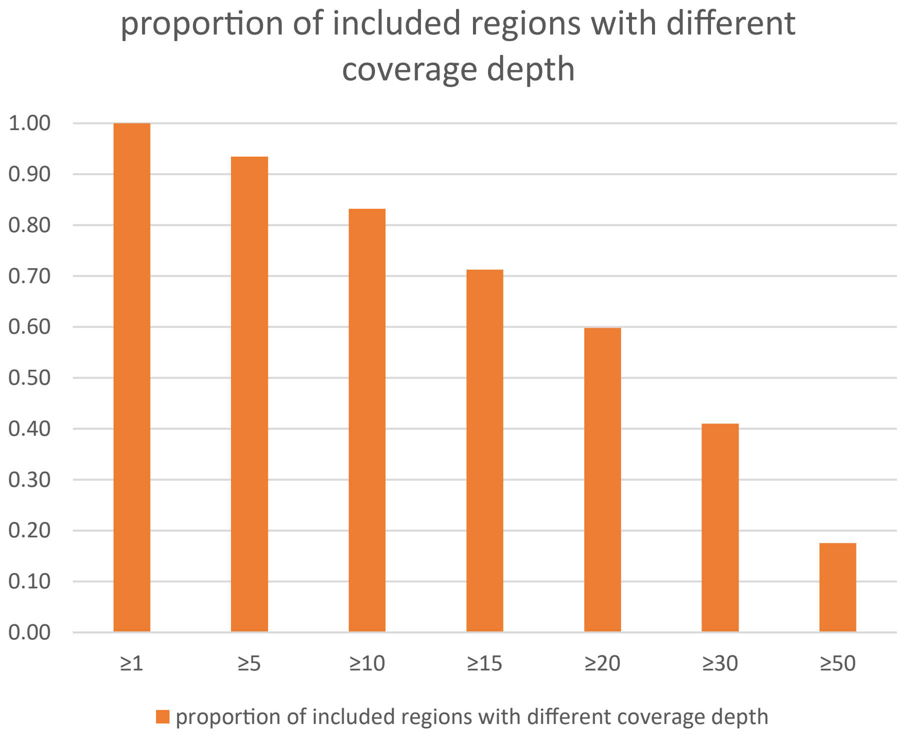

Supplementary Figure 3. The proportion of included regions with different coverage depth thresholds. The total region size of using coverage depth threshold ≥1 for at least 90% of samples is set as the reference.

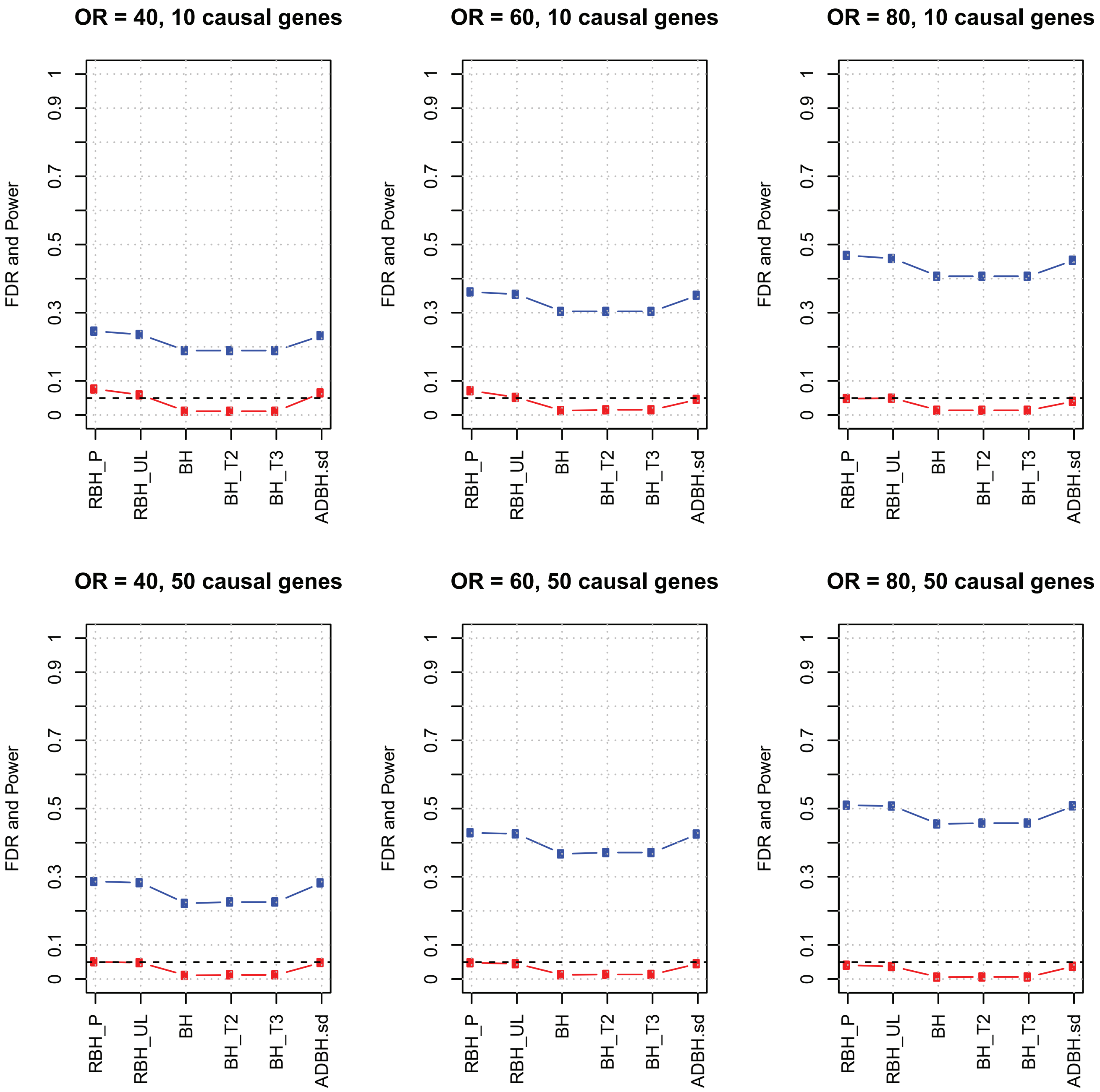

Supplementary Figure 4. Empirical FDR and power of different methods under different simulation settings. The performance under different combinations of the odds ratio (OR) and the number of causal genes was evaluated. The red color indicates empirical FDRs, the blue color indicates empirical powers. RBH_P: resampling based p-value adjustment using the point estimation; RBH_UL: resampling based p-value adjustment using the upper limit estimation; BH: the Benjamini Hochberg FDR control procedure; BH_T2, BH_T3: methods that remove genes with rare allele counts less than 2 or 3 and then apply the BH procedure; ADBH.sd: A-DBH-SD method from the R package DiscreteFDR. All tests are based on the two-sided Fisher's exact test.

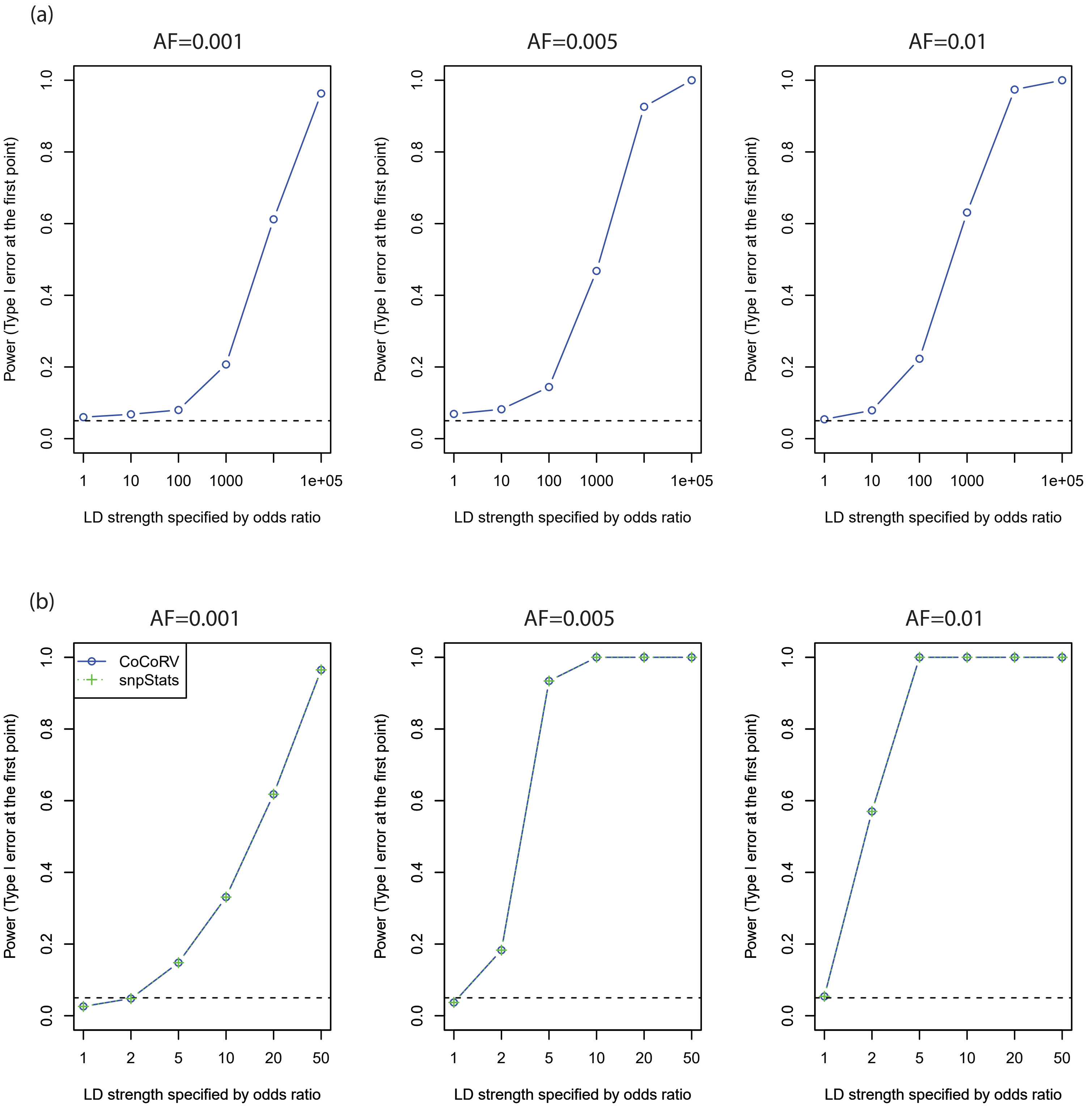

Supplementary Figure 5. Type I error and power of the proposed LD detection. **a.** Type I error and power of detecting LDs between rare variants based on six independent groups of summary counts using CoCoRV. The results are based on raw p-values from the one-sided likelihood ratio test (alternative hypothesis is high LD). **b**. Type I error and power of detecting LDs between rare variants based on full genotypes using CoCoRV and snpStats. The results are based on raw p-values with the two-sided likelihood ratio test because snpStats only outputs the two-sided test result. Type I error corresponds to the power when the odds ratio is 1. The nominal p-value threshold is 0.05, as indicated by the horizontal dashed line. AF, alternate allele frequency.

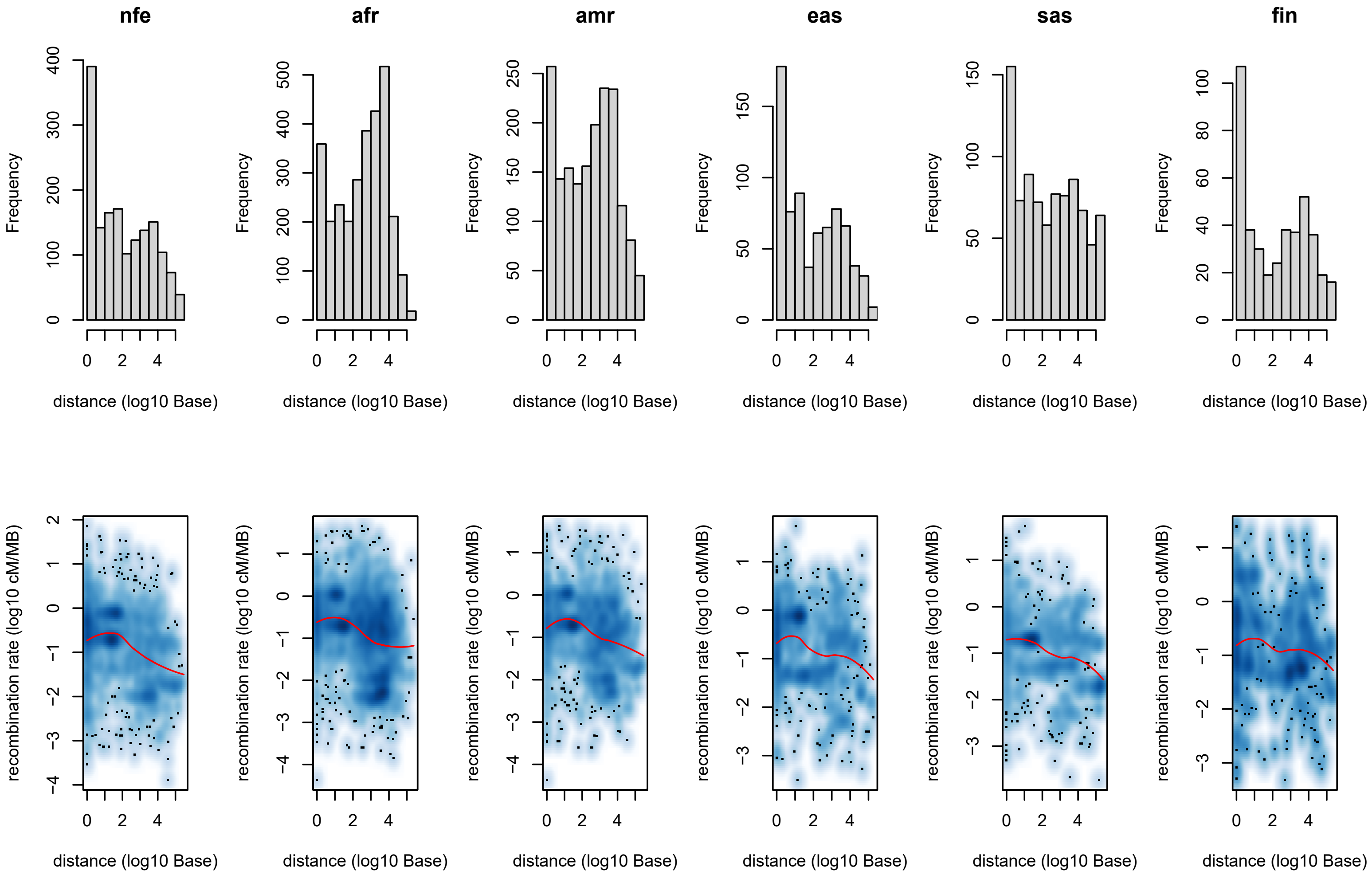

Supplementary Figure 6. Histograms of the distances of detected high-LD variants in gnomAD and the scatter plot of recombination rates versus the base-pair distances of detected high-LD variants in gnomAD. The distance between two variants is calculated based on the variants' positions after variant normalization. The upper panel shows the histograms of distances among different ethnicity groups. The lower panel shows the density of scatter plots between the recombination rates and the distances. The recombination rate is based on the middle point between two variants, and it is calculated based on linear interpolation using the genetic map downloaded from the Impute2 website (https://mathgen.stats.ox.ac.uk/impute/impute_v2.html#reference). The red line shows the nonlinear LOWESS fit. Abbreviations: nfe, non-Finnish European; afr, African American; amr, Admixed American; eas, East Asian; sas, South Asian; fin, Finnish; cM, centimorgan; MB, mega base-pairs.

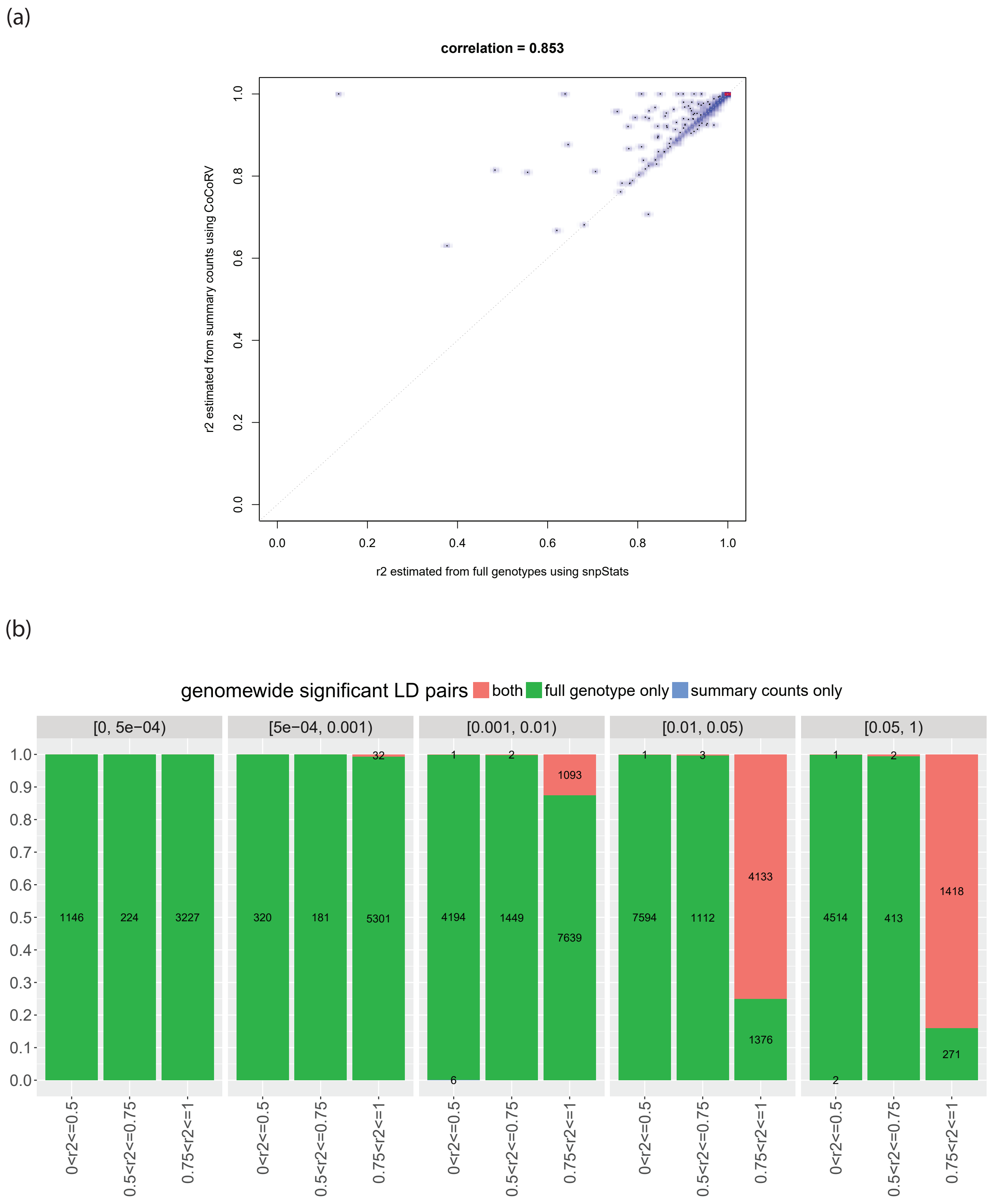

Supplementary Figure 7. Comparison of LD detection between using pooled summary counts and individual genotype on the inhouse control data set. a. comparison of LD measure r^2 with FDR < 0.05 in both methods. **b**. comparison of detected LD pairs stratified by the alternate allele frequency (the larger alternate allele frequency between a pair of variants in the control data set) and the estimated r^2 from using full genotypes.

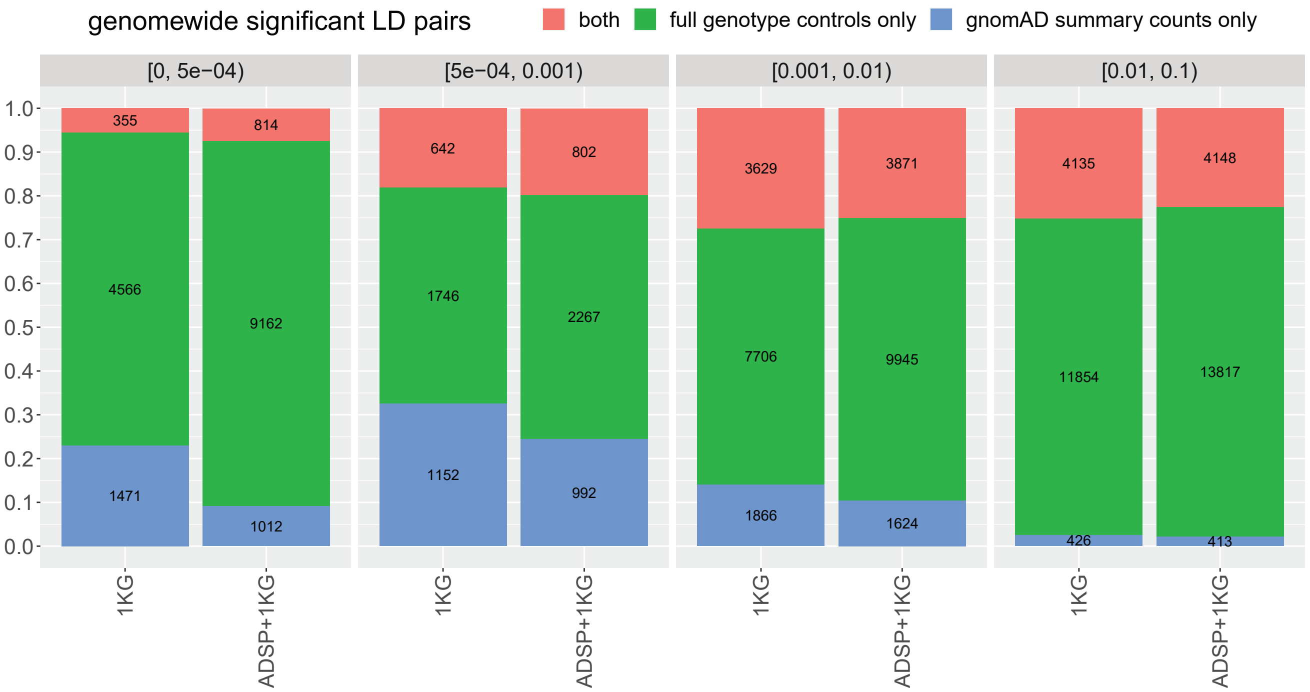

Supplementary Figure 8. The proportions of detected high LD pairs based on gnomAD summary counts and full genotype controls. There are two full genotype controls: samples from the 1,000 Genomes Project (1KG) and the constructed 8,175 controls (ADSP + 1KG). The y-axis shows the proportions from each subset.

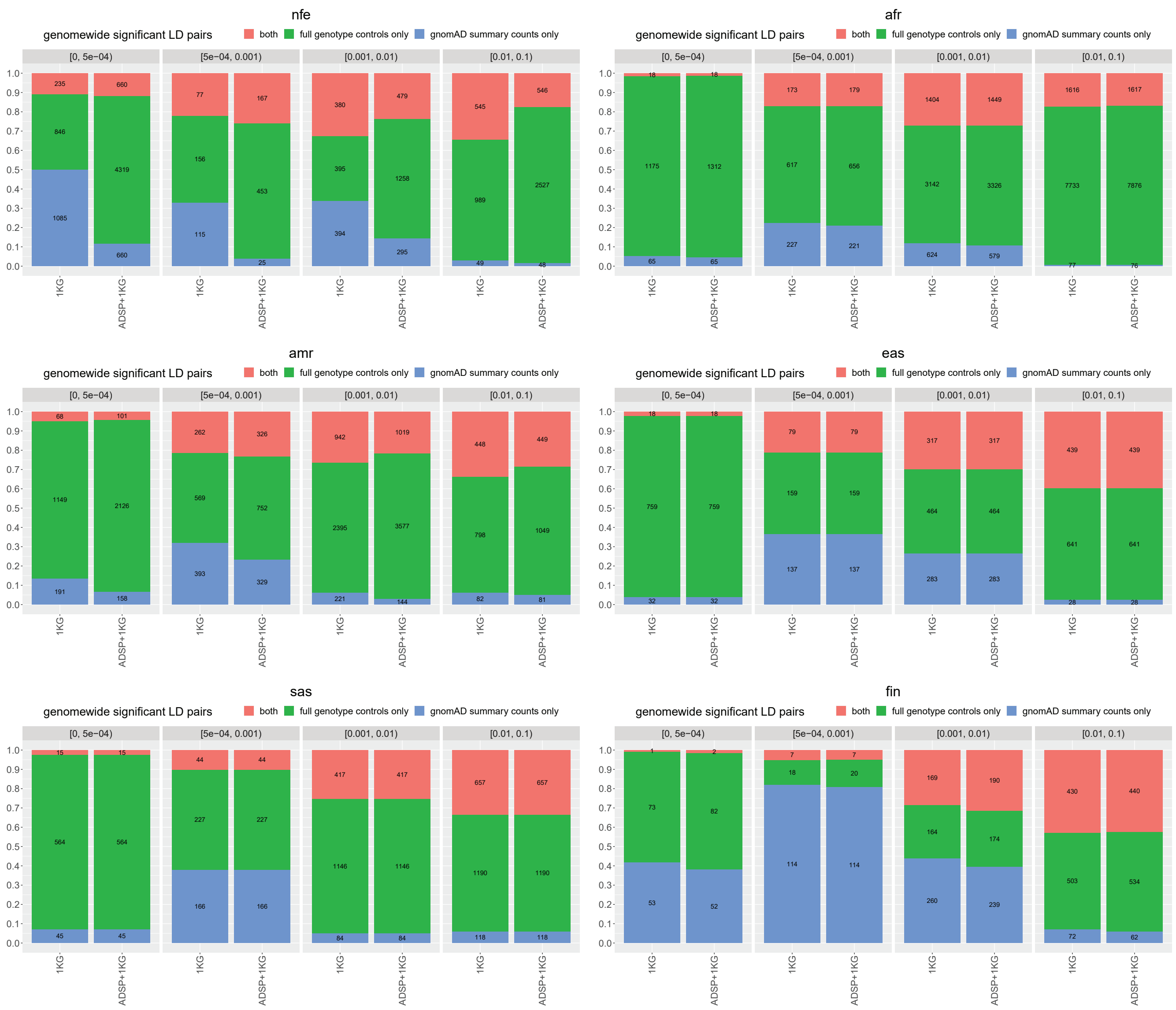

Supplementary Figure 9. The proportions of detected high LD pairs based on gnomAD summary counts and full genotype controls stratified by ethnicities. There are two full genotype controls: samples from the 1,000 Genomes Project (1KG) and the constructed 8,175 controls (ADSP + 1KG). The y-axis shows the proportions from each subset. Abbreviations: nfe, non-Finnish European; afr, African American; amr, Admixed American; eas, East Asian; sas, South Asian; fin, Finnish.

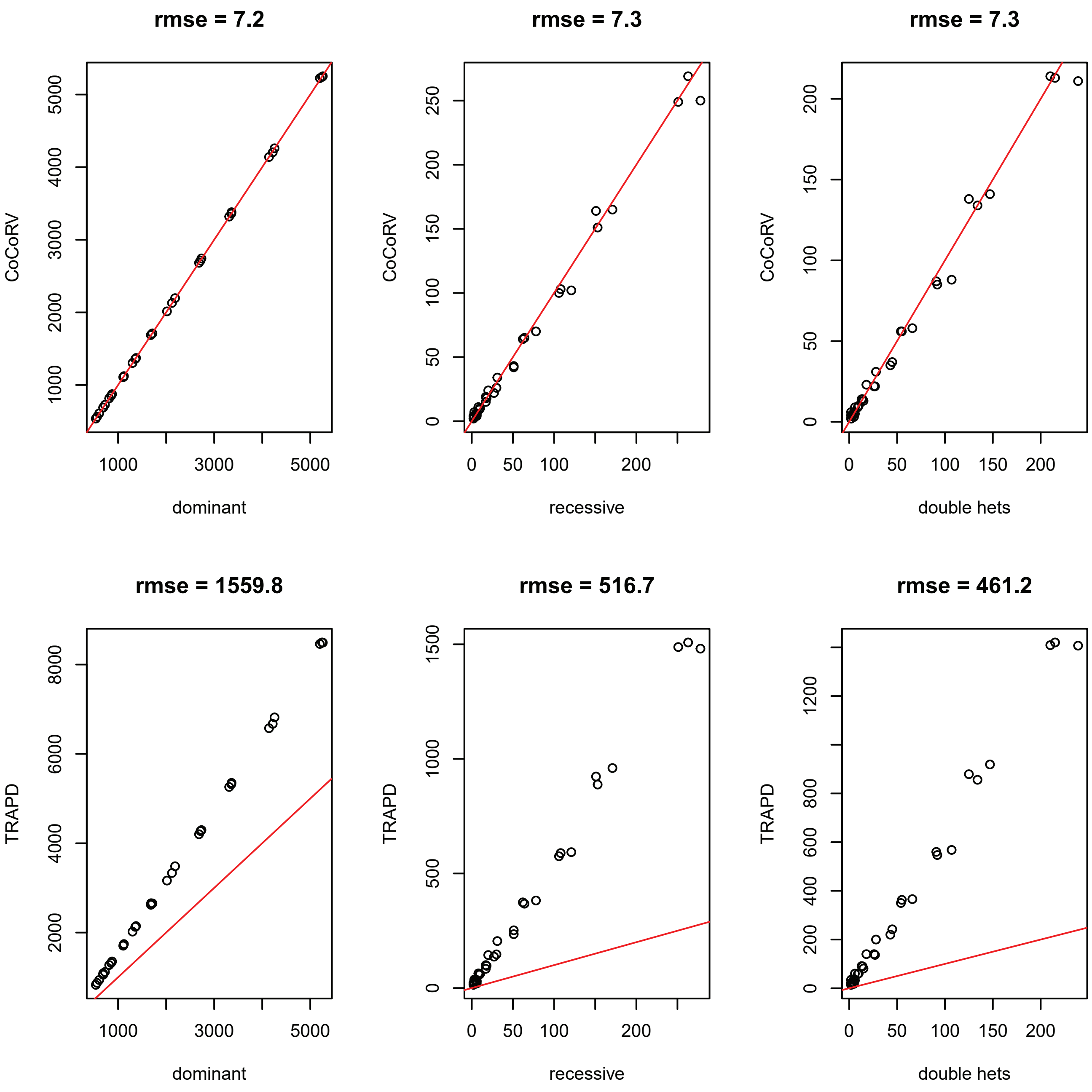

Supplementary Figure 10. Comparisons of count estimations under different models between CoCoRV and TRAPD. Top panels show the estimates from CoCoRV under the three different models (dominant, recessive, and double-heterozygous), and the bottom panels show those from TRAPD under the three models. The red lines are the $y = x$ lines; rmse, root mean squared error.

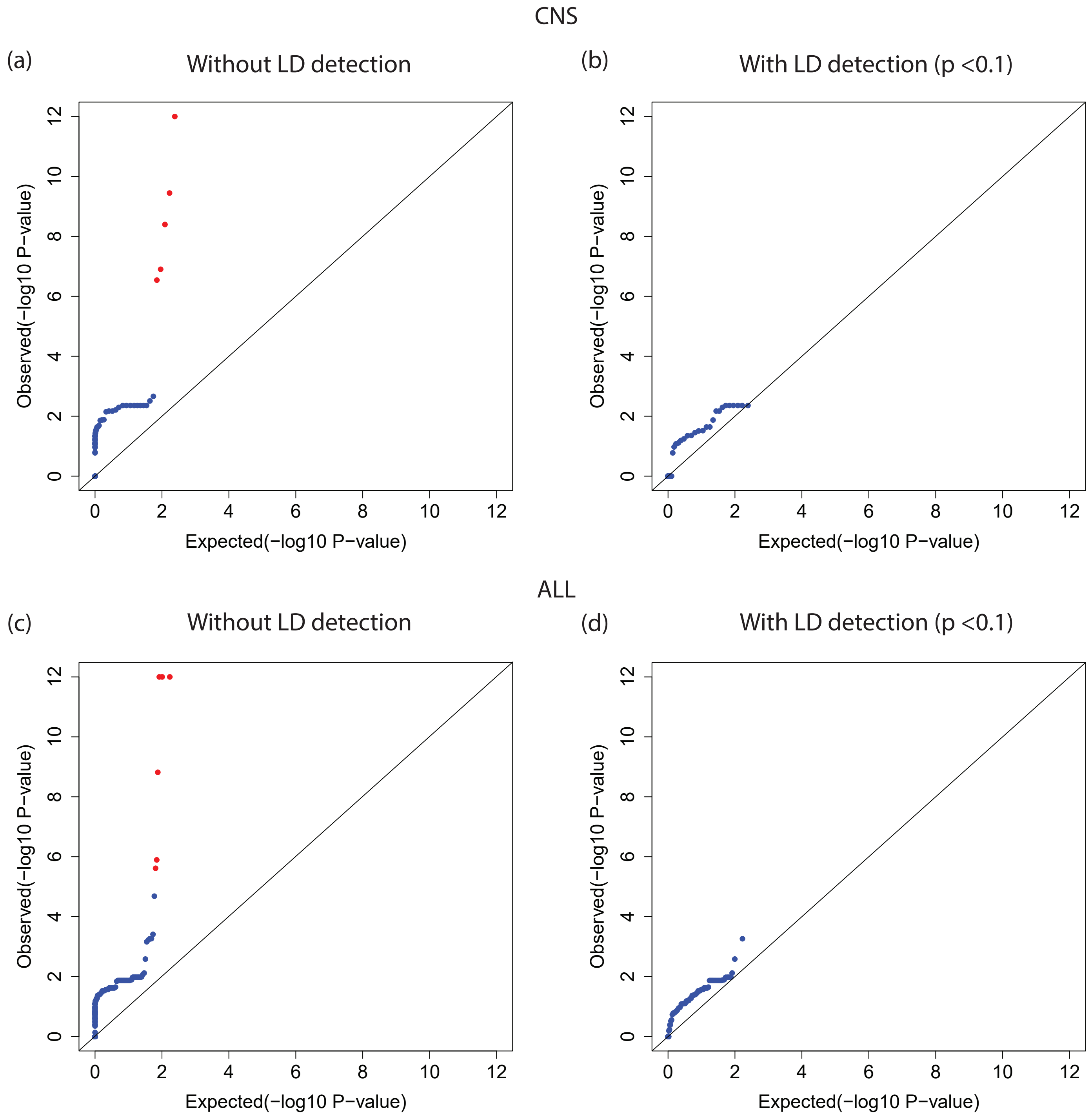

Supplementary Figure 11. Employing LD detection removes false positives under the double-heterozygous model. **a, c.** QQ plots of association tests under the double-heterozygous model without employing an LD test in the CNS (**a**) and ALL (**c**) cohorts. **b, d.** QQ plots of association tests after employing LD detection with a p-value threshold of 0.1 in the CNS (**b**) and ALL (**d**) cohorts. The p-values are raw p-values from the two-sided Cochran–Mantel–Haenszel (CMH) exact test.

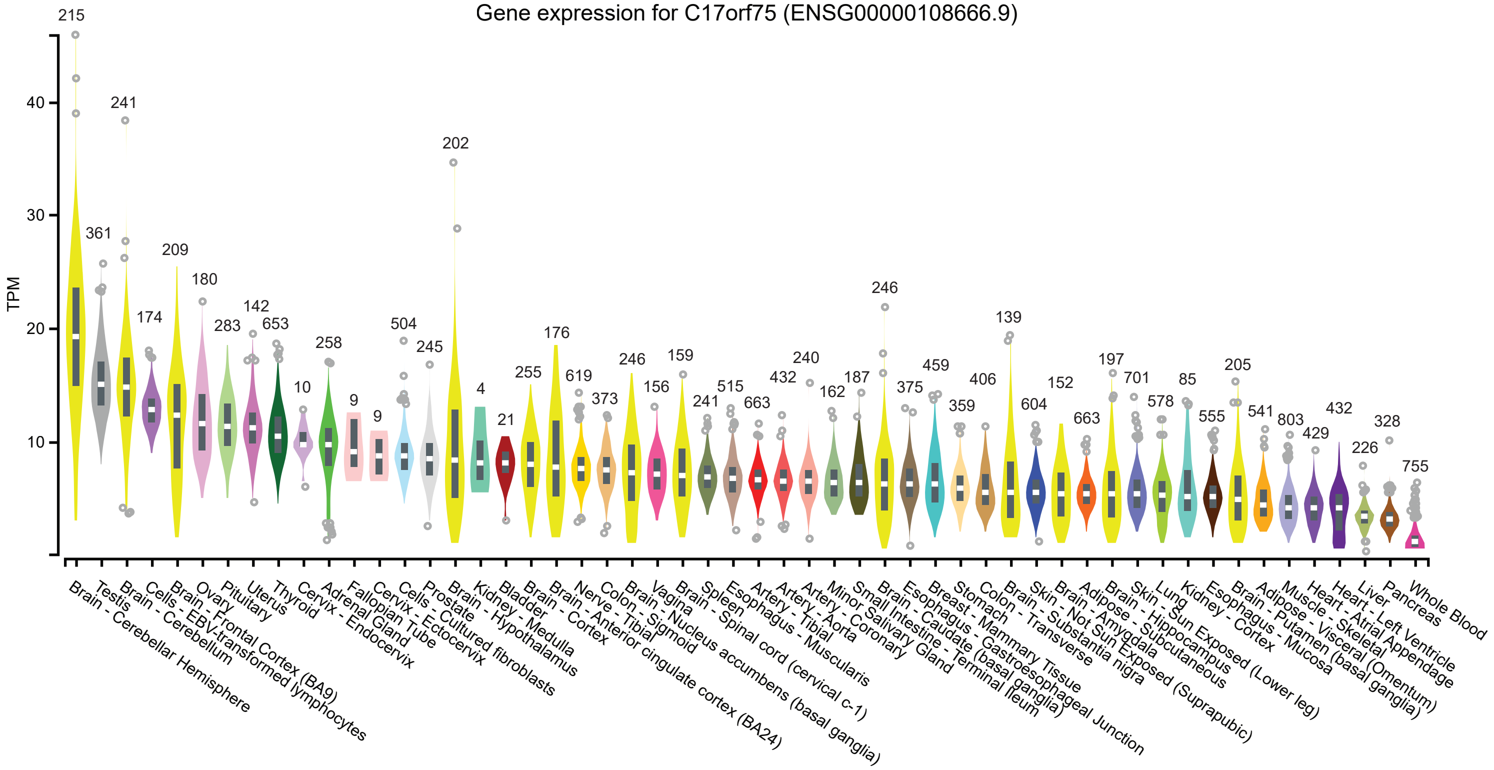

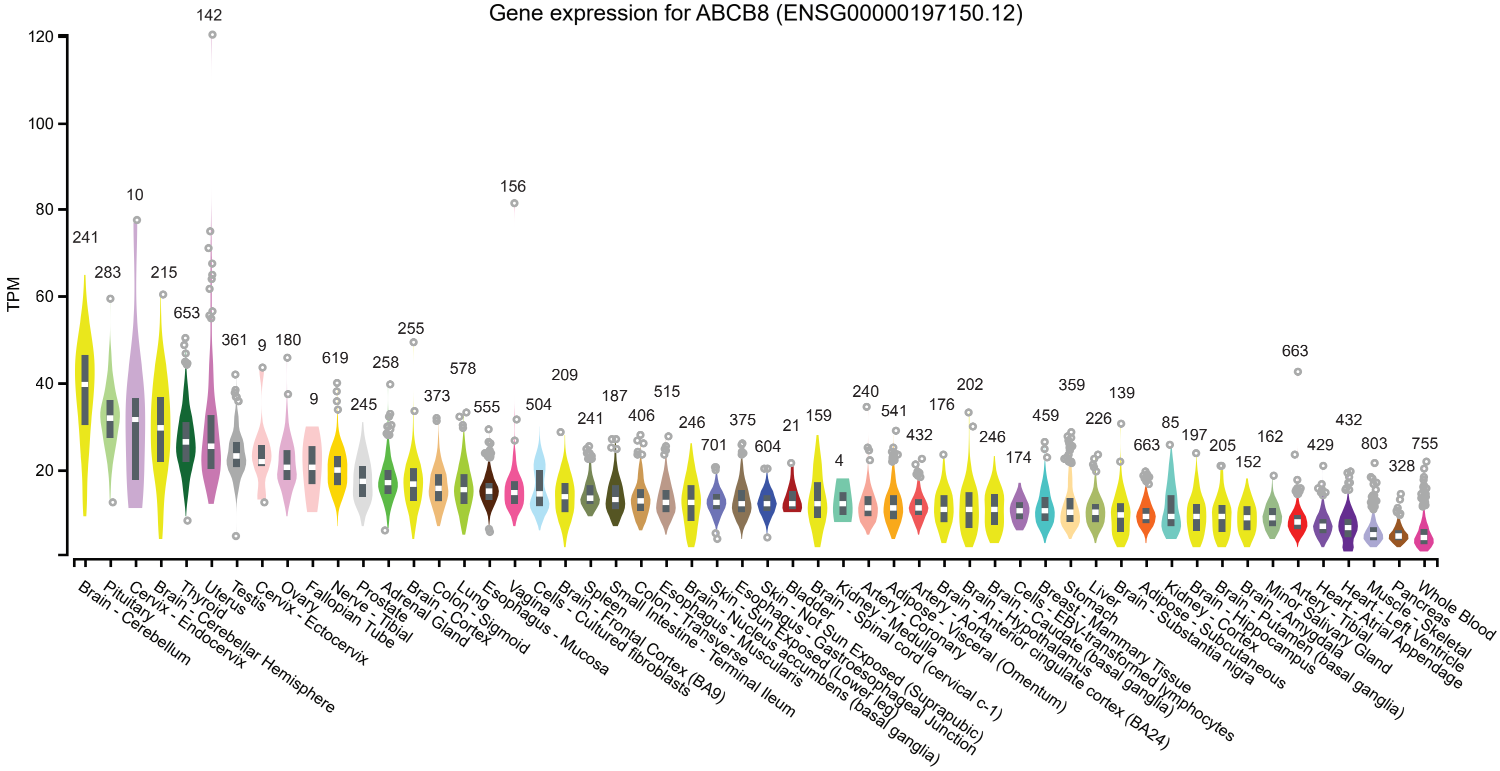

Supplementary Figure 12. The expression of *C17orf75* and *ABCB8* genes in different tissues from GTEx. Data obtained from the GTEx portal. The sample sizes of different tissues are shown on top of each subplot. For each subplot, the center is the median, the bounds of box covers between 25th and 75th percentile. Points are displayed as outliers if they are above or below 1.5 times the interquartile range starting from the 75th or 25th percentile. The minima is the bottom of the plot if there is no outlier at the bottom, otherwise it is the lowest outlier. The maxima is the top of the plot if there is no outlier at the top, otherwise it is the largest outlier.

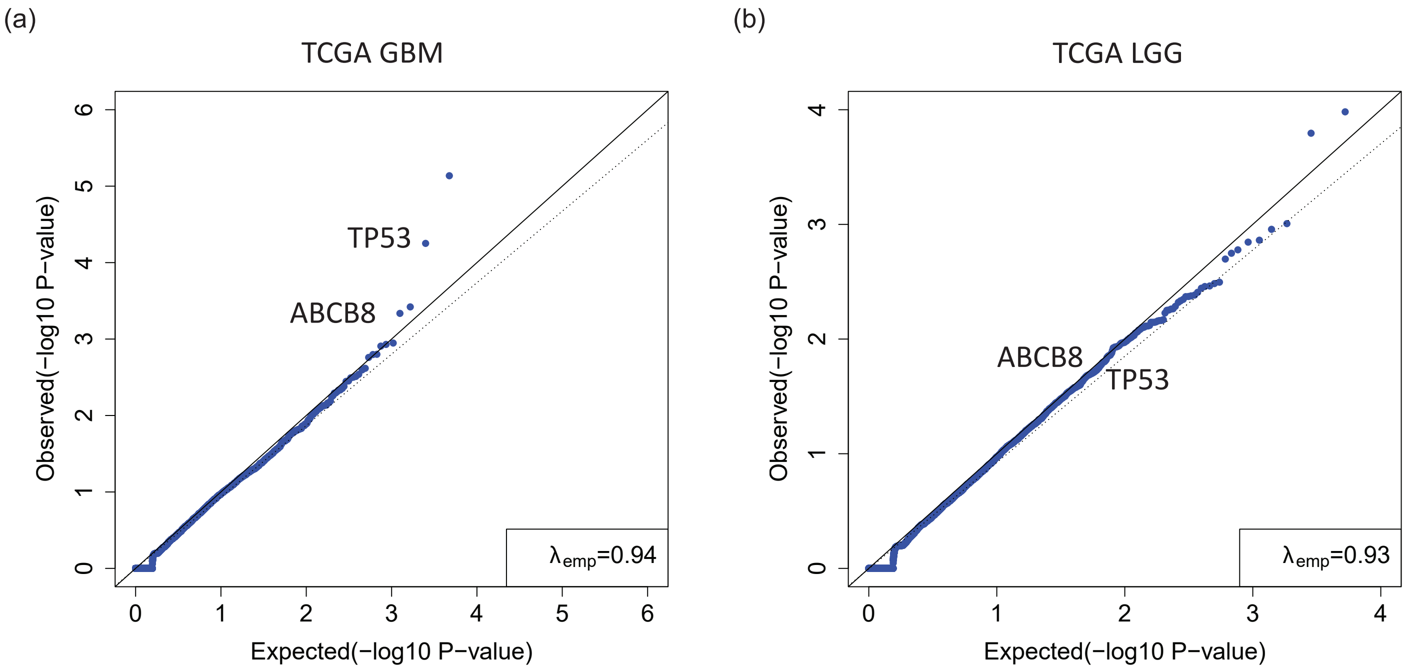

Supplementary Figure 13. QQ plots of the association tests for the TCGA GBM cohort and TCGA LGG cohort using the CoCoRV framework with gnomAD summary counts as controls. (a). QQ plot of the association results of the TCGA GBM cohort. (**b**)**.** QQ plot of association results of the TCGA LGG cohort. The p-values are raw p-values from the two-sided Cochran–Mantel–Haenszel (CMH) exact test.

(a)

Different ethnicities in the case and control samples						
	CNS	ALL	TCGA GBM	TCGA LGG	ADSP+1KG	gnomAD
nfe	250	776	235	356	4912	56885
fin	0	0	1	1	123	10824
afr	55	86	32	19	697	8128
amr	12	45	10	24	548	17296
eas	0	5	2	7	513	9197
sas	1	1	4	4	510	15308
other	18	45	41	72	872	3070

(b)

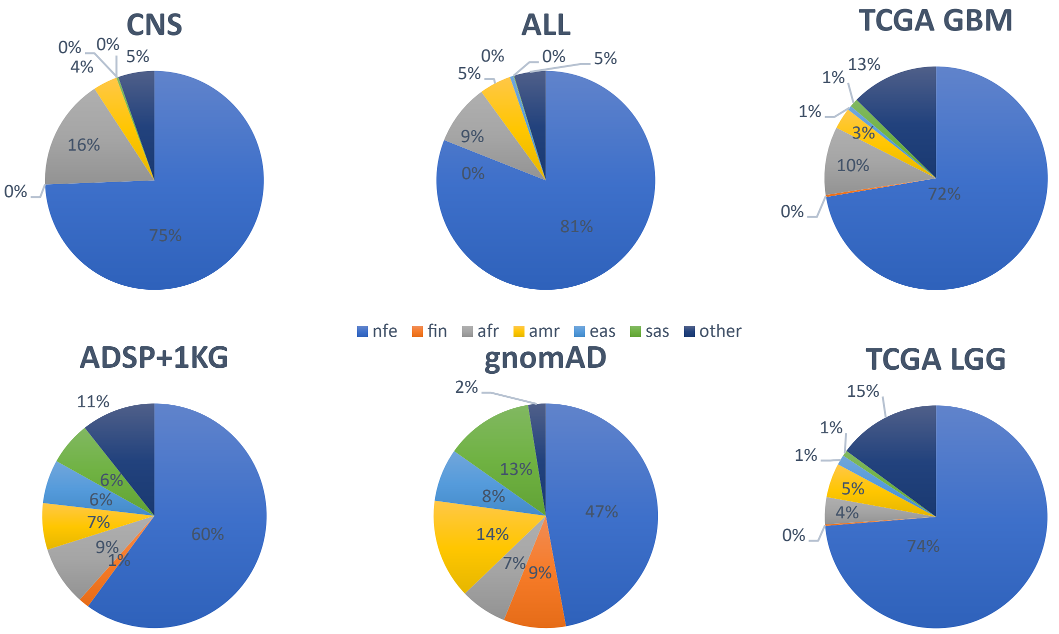

(c)

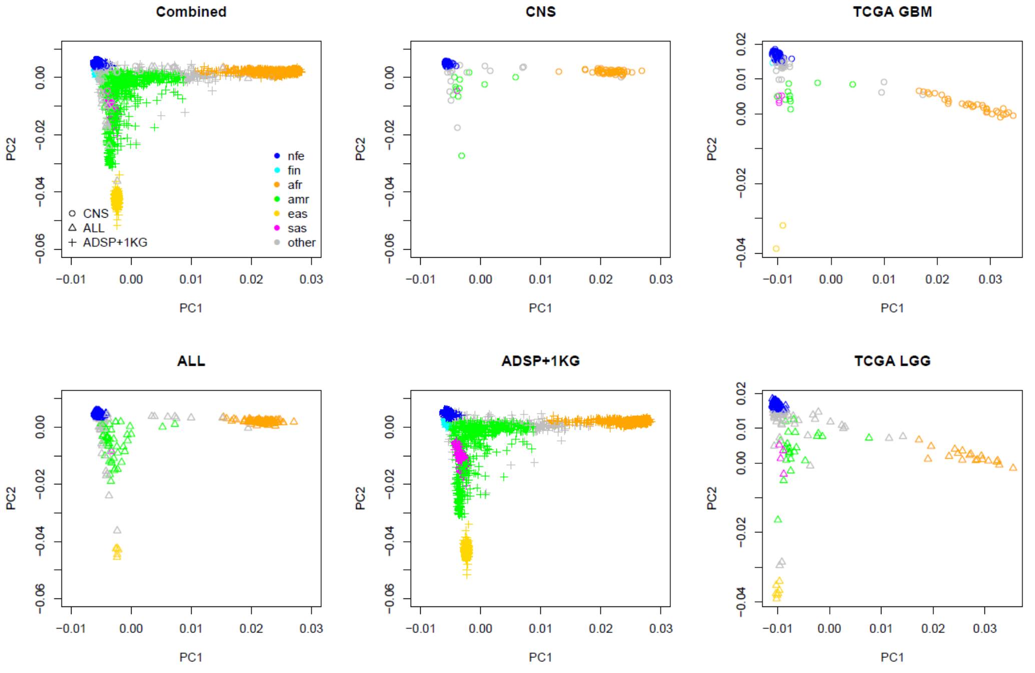

Supplementary Figure 14. Population structure of the data in our main analyses. Data sets included the CNS and ALL pediatric cancer cohorts and constructed in-house controls with samples from the Alzheimer's Disease Sequencing Project (ADSP), the 1,000 Genomes Project (1KG), and the gnomAD, TCGA GBM and TCGA LGG. **(a).** The number of samples in each ethnicity. **(b).** Pie charts showing the percentage of each ethnicity in each data set. **(c).** Plots of the top two principal components of the CNS, ALL, in-house controls, TCGA GBM and LGG. Abbreviations: nfe, non-Finnish European; afr, African American; amr, Admixed American; eas, East Asian; sas, South Asian; fin, Finnish; ALL, acute lymphoblastic leukemia; CNS, central nervous system cancer

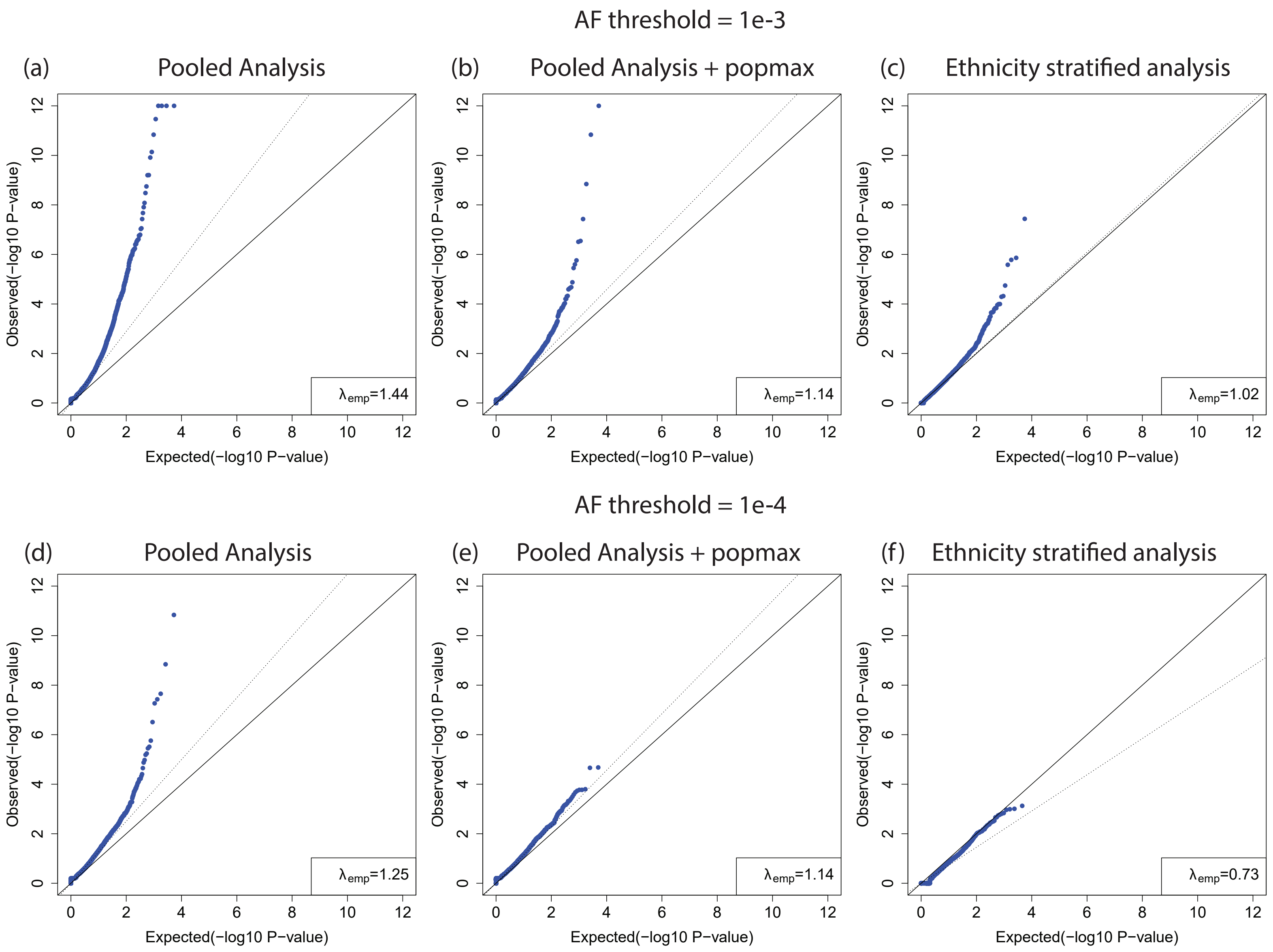

Supplementary Figure 15. QQ plots of pooled analysis and ethnicity stratified analysis where the ethnicity compositions of cases are very different from gnomAD. The "cases" are selected from the 1,000 Genomes Project covering five different ethnicities (nfe: 210, afr: 250, amr: 65, eas: 213, sas: 102). The y-axis is capped at 12. The raw p-values from the two-sided Fisher's exact test are used in (a-b) and (d-e). The raw p-values from the two-sided Cochran–Mantel–Haenszel (CMH) exact test are used in (c) and (f).

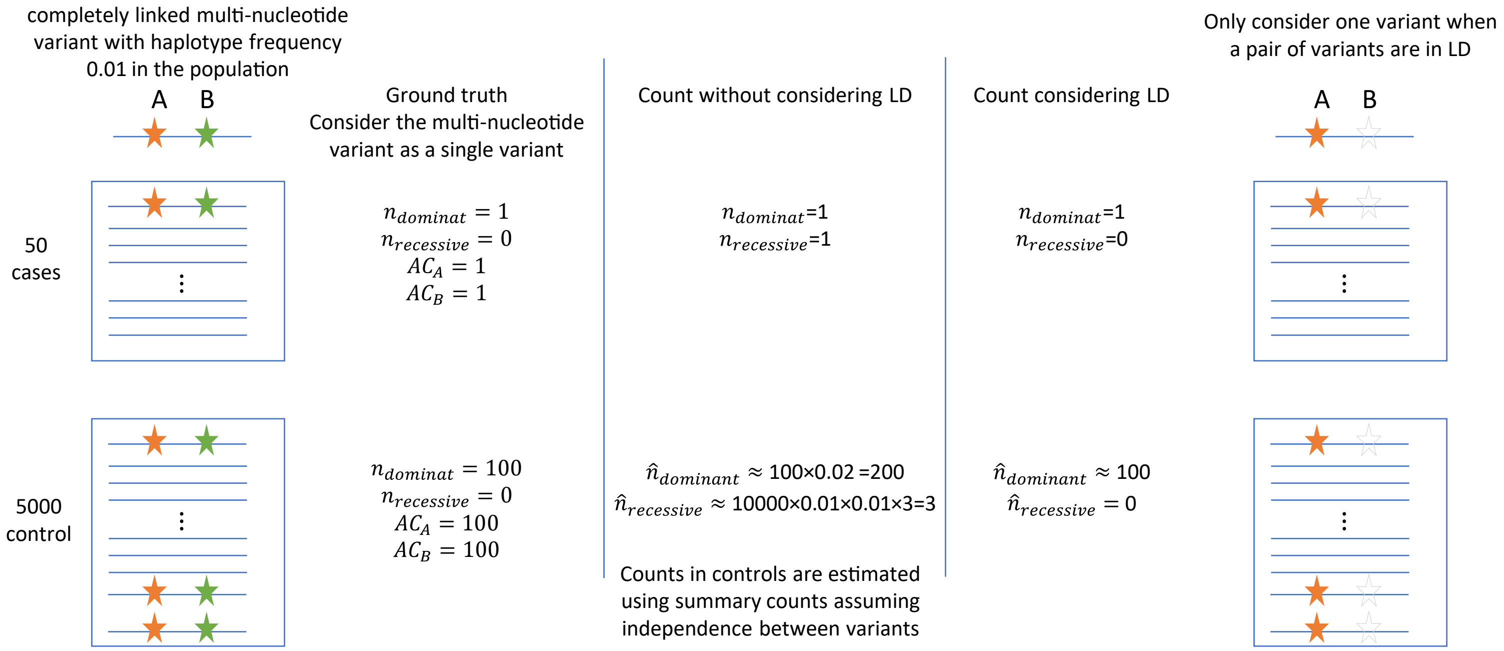

Supplementary Figure 16. An example illustrating the counting of samples under the dominant and recessive models with and without considering LD.

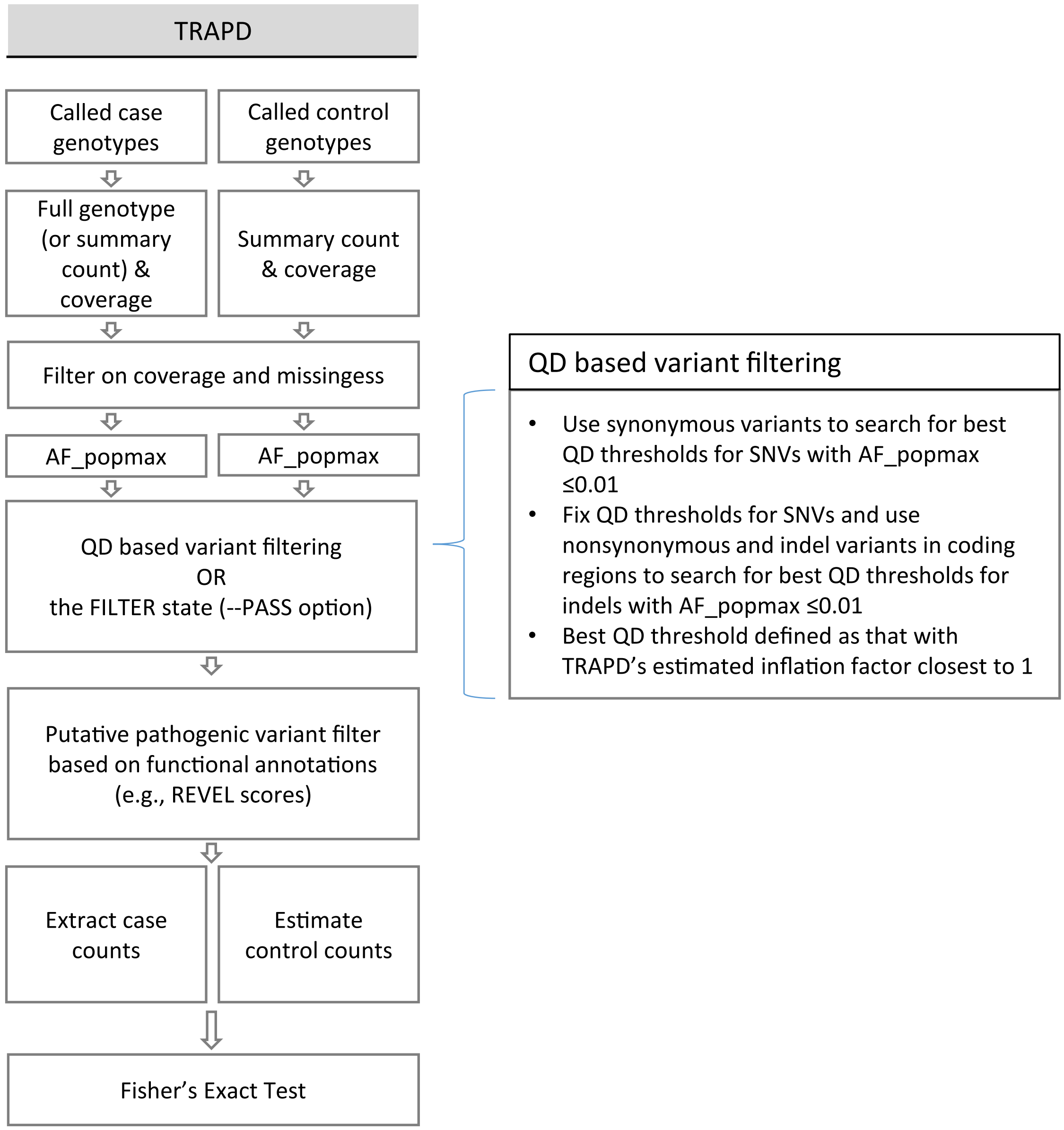

Supplementary Figure 17. Diagram of TRAPD analysis applied in the study.
