# Supplementary material for: A rare variant analysis framework using public genotype summary counts to prioritize disease-predisposition genes
Source: Nat Commun. 2022 May 11;13:2592. doi: 10.1038/s41467-022-30248-0 (PMC9095601; doi:10.1038/s41467-022-30248-0)
Supplement: Supplementary file 3 — Description of Additional Supplementary Files [file 41467_2022_30248_MOESM3_ESM.pdf]

Description of Additional Supplementary Files

File Name: Supplementary Data 1
Description: Summary of features among different tools.

File Name: Supplementary Data 2
Description: Concordance between separately called summary counts filtered by different coverage depths and jointly called case-control full genotypes

File Name: Supplementary Data 3
Description: Power of different methods to account for multiple testing.

File Name: Supplementary Data 4
Description: Adjusted P-values of different methods of the SJLIFE CNS association test result using gnomAD as the controls.

File Name: Supplementary Data 5
Description: Detected high LD variants in gnomAD exomes with FDR < 0.05 in each ethnicity group.

File Name: Supplementary Data 6
Description: Specificity and recall of the LD test when using gnomAD MNVs (BP<=2) as the ground truth.

File Name: Supplementary Data 7
Description: Variants in high LD that causes the false positives under the recessive model.

File Name: Supplementary Data 8
Description: False positives and true positives of different methods under the dominant model using gnomAD summary counts as the controls.

File Name: Supplementary Data 9
Description: Comparison of SJLIFE counts and p-values between CoCoRV sample count based analysis and ProxECAT.

File Name: Supplementary Data 10
Description: False positives and true positives of different methods under the recessive models using gnomAD summary counts as the controls.

File Name: Supplementary Data 11
Description: False positives and true positives of different methods under the dominant models using gnomAD summary counts as the controls and a subset of 1000 Genomes as simulated "cases".

File Name: Supplementary Data 12
Description: The top false positive genes and the driving variants using gnomAD summary counts as the controls and a subset of 1000 Genomes as simulated "cases”.
