# Supplementary material for: A rare variant analysis framework using public genotype summary counts to prioritize disease-predisposition genes
Source: Nat Commun. 2022 May 11;13:2592. doi: 10.1038/s41467-022-30248-0 (PMC9095601; doi:10.1038/s41467-022-30248-0)
Supplement: Supplementary file 5 — Reporting Summary [file 41467_2022_30248_MOESM5_ESM.pdf]

nature portfolio

Corresponding author(s): Wenan Chen, Gang Wu

Last updated by author(s): Mar 29, 2022

Reporting Summary

Nature Portfolio wishes to improve the reproducibility of the work that we publish. This form provides structure for consistency and transparency in reporting. For further information on Nature Portfolio policies, see our Editorial Policies and the Editorial Policy Checklist.

Statistics

For all statistical analyses, confirm that the following items are present in the figure legend, table legend, main text, or Methods section.

n/a	Confirmed	
☐	☒	The exact sample size (*n*) for each experimental group/condition, given as a discrete number and unit of measurement
☒	☐	A statement on whether measurements were taken from distinct samples or whether the same sample was measured repeatedly
☐	☒	The statistical test(s) used AND whether they are one- or two-sided<br>*Only common tests should be described solely by name; describe more complex techniques in the Methods section.*
☒	☐	A description of all covariates tested
☐	☒	A description of any assumptions or corrections, such as tests of normality and adjustment for multiple comparisons
☐	☒	A full description of the statistical parameters including central tendency (e.g. means) or other basic estimates (e.g. regression coefficient) AND variation (e.g. standard deviation) or associated estimates of uncertainty (e.g. confidence intervals)
☐	☒	For null hypothesis testing, the test statistic (e.g. *F*, *t*, *r*) with confidence intervals, effect sizes, degrees of freedom and *P* value noted<br>*Give P values as exact values whenever suitable.*
☒	☐	For Bayesian analysis, information on the choice of priors and Markov chain Monte Carlo settings
☒	☐	For hierarchical and complex designs, identification of the appropriate level for tests and full reporting of outcomes
☐	☒	Estimates of effect sizes (e.g. Cohen's *d*, Pearson's *r*), indicating how they were calculated

Our web collection on statistics for biologists contains articles on many of the points above.

Software and code

Policy information about availability of computer code

Data collection	BWA v0.7.12, GATK v3.7, samtools 1.10
Data analysis	bcftools v1.9, plink 1.9, R 4.0.0, python 3, snpStats v1.3.8, EPACTS v3.3.0, GENESIS v2.4.0, TRAPD (last commit 11/15/2019), ProXECAT 0.2.0, CoCoRV v1.0 (https://bitbucket.org/Wenan/cocorv/src/master/), BiasedUrn v1.07, ANNOVAR (2017-07-17), picard v2.21.2

For manuscripts utilizing custom algorithms or software that are central to the research but not yet described in published literature, software must be made available to editors and reviewers. We strongly encourage code deposition in a community repository (e.g. GitHub). See the Nature Portfolio guidelines for submitting code & software for further information.

Data

Policy information about availability of data

All manuscripts must include a data availability statement. This statement should provide the following information, where applicable:

- Accession codes, unique identifiers, or web links for publicly available datasets
- A description of any restrictions on data availability
- For clinical datasets or third party data, please ensure that the statement adheres to our policy

GRCh37-lite is available here: ftp://ftp.ncbi.nih.gov/genomes/archive/old_genbank/Eukaryotes/vertebrates_mammals/Homo_sapiens/GRCh37/special_requests/GRCh37-lite.fa.gz
UCSC CRG Align 36 track is available here: http://hgdownload.cse.ucsc.edu/goldenpath/hg19/encodeDCC/wgEncodeMapability/wgEncodeCrgMapabilityAlign36mer.bigWig
The gnomAD summary count data is available here: https://gnomad.broadinstitute.org/
The gnomAD detected MNV is available here: https://gnomad.broadinstitute.org/downloads#v2-multi-nucleotide-variants

The TOPMed summary count data is available here: https://bravo.sph.umich.edu/
ALS data is available here: http://alsdb.org/downloads
Alzheimer's Disease Sequencing Project (ADSP) with restricted access: https://www.ncbi.nlm.nih.gov/projects/gap/cgi-bin/study.cgi?study_id=phs000572.v8.p4
1,000 Genomes Project is available here: https://www.internationalgenome.org/
The Cancer Genome Atlas (TCGA) with restricted access: https://www.ncbi.nlm.nih.gov/projects/gap/cgi-bin/study.cgi?study_id=phs000178.v11.p8
St Jude cancer cohort with restricted access: https://www.stjude.cloud/
The GTEx portal is available here: https://gtexportal.org/home

Field-specific reporting

Please select the one below that is the best fit for your research. If you are not sure, read the appropriate sections before making your selection.

- [x] Life sciences
- [ ] Behavioural & social sciences
- [ ] Ecological, evolutionary & environmental sciences

For a reference copy of the document with all sections, see nature.com/documents/nr-reporting-summary-flat.pdf

Life sciences study design

All studies must disclose on these points even when the disclosure is negative.

Sample size	No sample size calculation, we try to use as many samples as possible to prioritize risk genes
Data exclusions	Data failed sequencing quality control are excluded, such as the gentoype missingness within an individual is too high indicating pool gentoype quality. Related samples are also excluded
Replication	For the two top prioritized genes TP53 and ABCB8 in TCGA, two genes can be replicated in the PCGP cohort. The two genes cannot be replicated in the SJLIFE CNS cohort, a childhood cancer survival cohort. Other three genes out of the top five genes from the TCGA GBM cohort are not pursued for replication because they are not significant in the TCGA LGG cohort.
Randomization	No randomization is used, we use all available samples that we can access. Another reason why randomization is not used is that for genetic associations, the major confounding factor is the population structure/ancestry which can be accounted for in the analysis, e.g., by including the top principal components when the individual genotype data is available. In our analysis, when only summary counts are available for the controls, we use ethnicity-stratified analysis to ameliorate the confounding effect of population structure.
Blinding	The phenotype genotype association is blind because when the phenotype/cancer type is assigned, the genotype information is not used. For all other data collection/analysis, we try to make all processing/QC/filtering as consistent as possible between cases and controls, and the underlying collection/analysis is blind to the underlying genotypes.

Reporting for specific materials, systems and methods

We require information from authors about some types of materials, experimental systems and methods used in many studies. Here, indicate whether each material, system or method listed is relevant to your study. If you are not sure if a list item applies to your research, read the appropriate section before selecting a response.

Materials & experimental systems

n/a	Involved in the study	
x		Antibodies
x		Eukaryotic cell lines
x		Palaeontology and archaeology
x		Animals and other organisms
x		Human research participants
x		Clinical data
x		Dual use research of concern

Methods

n/a	Involved in the study	
x		ChIP-seq
x		Flow cytometry
x		MRI-based neuroimaging
